# Predictive performance of automated surveillance algorithms for intravascular catheter bloodstream infections: a systematic review and meta-analysis

**DOI:** 10.1186/s13756-023-01286-0

**Published:** 2023-08-31

**Authors:** Jean-Marie Januel, Nasim Lotfinejad, Rebecca Grant, Sarah Tschudin-Sutter, Peter W. Schreiber, Bruno Grandbastien, Philipp Jent, Elia Lo Priore, Alexandra Scherrer, Stephan Harbarth, Gaud Catho, Niccolò Buetti, Swissnoso, Carlo Balmelli, Delphine Berthod, Jonas Marschall, Hugo Sax, Matthias Schlegel, Alexander Schweiger, Laurence Senn, Rami Sommerstein, Nicolas Troillet, Danielle Vuichard Gysin, Andreas F Widmer, Aline Wolfensberger, Walter Zingg

**Affiliations:** 1 Infection Control Program and WHO Collaborating Centre, Geneva University Hospitals and Faculty of Medicine, Service PCI, Rue Gabrielle-Perret-Gentil 4, 1205 Geneve, Switzerland; 2 Division of Infectious Diseases & Hospital Epidemiology, University Hospital Basel and University of Basel, Basel, Switzerland; 3 Department of Infectious Diseases and Hospital Epidemiology, University Hospital Zurich and University of Zurich, Zurich, Switzerland; 4 Service of Hospital Preventive Medicine, Lausanne University Hospital, Lausanne, Switzerland; 5 Department of Infectious Diseases, Bern University Hospital, University of Bern, Bern, Switzerland; 6 Department of Infectious Diseases and Hospital Epidemiology, EOC Regional Hospital of Lugano, Lugano, Switzerland; 7 Swissnoso, National Center for Infection Control, Bern, Switzerland; 8 Division of Infectious Diseases, Central Institute, Valais Hospital, Sion, Switzerland; 9 Université de Paris, INSERM, IAME UMR 1137, 75018 Paris, France

**Keywords:** CLABSI, CRBSI, Automated monitoring, Algorithm, Accuracy, Surveillance, Healthcare associated infections

## Abstract

**Background:**

Intravascular catheter infections are associated with adverse clinical outcomes. However, a significant proportion of these infections are preventable. Evaluations of the performance of automated surveillance systems for adequate monitoring of central-line associated bloodstream infection (CLABSI) or catheter-related bloodstream infection (CRBSI) are limited.

**Objectives:**

We evaluated the predictive performance of automated algorithms for CLABSI/CRBSI detection, and investigated which parameters included in automated algorithms provide the greatest accuracy for CLABSI/CRBSI detection.

**Methods:**

We performed a meta-analysis based on a systematic search of published studies in PubMed and EMBASE from 1 January 2000 to 31 December 2021. We included studies that evaluated predictive performance of automated surveillance algorithms for CLABSI/CRBSI detection and used manually collected surveillance data as reference. We estimated the pooled sensitivity and specificity of algorithms for accuracy and performed a univariable meta-regression of the different parameters used across algorithms.

**Results:**

The search identified five full text studies and 32 different algorithms or study populations were included in the meta-analysis. All studies analysed central venous catheters and identified CLABSI or CRBSI as an outcome. Pooled sensitivity and specificity of automated surveillance algorithm were 0.88 [95%CI 0.84–0.91] and 0.86 [95%CI 0.79–0.92] with significant heterogeneity (*I*^2^ = 91.9, *p* < 0.001 and *I*^2^ = 99.2, *p* < 0.001, respectively). In meta-regression, algorithms that include results of microbiological cultures from specific specimens (respiratory, urine and wound) to exclude non-CRBSI had higher specificity estimates (0.92, 95%CI 0.88–0.96) than algorithms that include results of microbiological cultures from any other body sites (0.88, 95% CI 0.81–0.95). The addition of clinical signs as a predictor did not improve performance of these algorithms with similar specificity estimates (0.92, 95%CI 0.88–0.96).

**Conclusions:**

Performance of automated algorithms for detection of intravascular catheter infections in comparison to manual surveillance seems encouraging. The development of automated algorithms should consider the inclusion of results of microbiological cultures from specific specimens to exclude non-CRBSI, while the inclusion of clinical data may not have an added-value.

* Trail Registration* Prospectively registered with International prospective register of systematic reviews (PROSPERO ID CRD42022299641; January 21, 2022). https://www.crd.york.ac.uk/prospero/display_record.php?ID=CRD42022299641

**Supplementary Information:**

The online version contains supplementary material available at 10.1186/s13756-023-01286-0.

## Background

Intravascular catheters (IVC) are indispensable and commonly used medical devices in hospitalized patients, and substantially predispose patients to develop healthcare-associated infections (HAIs) [1–9].

In 2016, the mean prevalence of HAIs in European countries was estimated to be 6.5% [1]. Hospital-acquired bloodstream infections (HA-BSI) account for 14.2% of HAI [2] and a large proportion of HA-BSI is attributable to IVC. In Europe, both central line-associated bloodstream infection (CLABSI) and catheter-related bloodstream infection (CRBSI) represent 36.5% of intensive care unit (ICU)-acquired bloodstream infections (BSIs) and the incidence has been found to vary between 1.7 and 4.8 episodes per 1000 catheter days [4]. CLABSI/CRBSI are associated with excess mortality rates, extended duration of hospitalization and greater healthcare expenditure [5–9]. More than 50% of CLABSI/CRBSI can be considered as preventable [3].

Surveillance of CLABSI/CRBSI among patients with IVC allows for the burden of disease to be quantified and for the effectiveness of interventions to prevent CLABSI/CRBSI to be assessed. Automated algorithms may offer approaches to improve the efficiency of CLABSI/CRBSI surveillance. Compared to manual surveillance, automated surveillance has been demonstrated to reduce time and workload for healthcare workers and infection control practitioners, and to provide data in real time that may allow for more timely clinical interventions [10–12]. However, little is known as to the predictive performance of automated algorithms for CLABSI/CRBSI surveillance, as well as the relative performance of different parameters that could be used within automated algorithms for the timely identification of CLABSI/CRBSI among patients with IVC.

The main objective of this study was to evaluate the predictive performance of automated surveillance systems for the detection of CLABSI/CRBSI among hospitalized patients, and to identify which parameters have a greater influence on the predictive performance, so as to inform future automated surveillance algorithms for CLABSI/CRBSI detection.

## Methods

### Design

We performed a systematic review and meta-analysis on the predictive performance of automated surveillance algorithms for the detection of CLABSI/CRBSI among hospitalized patients. This study was registered within the PROSPERO international prospective register of systematic reviews (CRD42022299641) on January 21, 2022, and was reported in accordance with the Preferred Reporting Items for Systematic reviews and Meta-Analyses (PRISMA) statement [13, 14].

### Search strategy

We conducted a systematic search using two electronic databases, PubMed and EMBASE, for relevant articles published between 1 January 2000 and 31 December 2021. We searched for original studies using the keyword algorithms described in the supplementary material. The search was limited to articles published in English. We searched for studies that reported on the predictive performance of automated algorithms for the detection of HAI (to increase the sensitivity of the search strategy) and of intravascular catheter infections (to increase the specificity of the search strategy). The records from the two databases search were merged and duplicates were removed using the EndNote program (Thomson Reuters, New-York, NY, USA).

### Study selection

Two investigators (J.M.J. and N.L.) screened titles and abstracts and examined the full text of original articles selected for study inclusion independently and in duplicate; disagreements were resolved by consensus.

### Inclusion criteria

Original studies were included if they assessed accuracy of automated algorithms for the surveillance of CLABSI and/or CRBSI. CLABSI was defined by one positive blood culture and clinical manifestation of infection in a patient with a catheter in place and with no other source of bacteremia except the catheter. CRBSI was defined as one positive blood culture obtained from peripheral vein and clinical manifestation of infection, and at least one of the following: (1) a positive CVC culture with the same micro-organism by qualitative or semi-quantitative (i.e., ≥ 15 CFU) methods or (2) a differential time to positivity of more than 120 min between central catheter blood culture and peripheral blood culture (blood samples drawn at the same time), or (3) a ratio of microorganism quantity from CVC blood sample on microorganism quantity from peripheral blood sample greater than 3 [15–17]. We selected studies that compared the predictive performance of automated algorithms with data from manual surveillance. Selected studies needed to include sensitivity and specificity estimates calculated using diagnostic test methods. Moreover, studies needed to directly or indirectly include all of the following: number of true positives (TP), false positives (FP), true negatives (TN), and false negatives (FN). Studies that did not provide all these data to determine the predictive performance of the automated algorithm were excluded.

### Data extraction and quality assessment

Data were extracted from the selected studies according to predefined rules that were used to identify IVC infections. If multiple algorithms (i.e., algorithms with different definitions for identifying IVC infections) or multiple study populations were evaluated in a single study, we defined each single algorithm as a single observation in the meta-analysis. The total number of algorithms analyzed was therefore higher than the number included studies. For each algorithm, we extracted the data on the endpoint (CLABSI/CRBSI) and on the predictive performance of the automated algorithm: TP, FP, FN, and TN.

From these different algorithms, we identified individual and pooled parameters for CLABSI/CRBSI detection.

Using the revised tool for the Quality Assessment of Diagnostic Accuracy Studies (QUADAS 2) [18], we evaluated the quality of studies based on four items: patient selection, index test, reference standard, and flow and timing. We assessed intra-study risk of bias and concern for applicability using a three-level rating scale (high, low or unclear).

### Statistical analysis

We first described characteristics of included studies (mono *versus* multicenter, type of catheter included, setting) and outcomes. We then estimated pooled sensitivity and specificity of automated surveillance algorithms for the identification of CLABSI/CRBSI with 95% confidence intervals (95% CI) for algorithms using bivariate random-effects models. We used *I*^2^ statistics to assess potential heterogeneity between algorithms [19, 20], with *I*^2^ > 75% representing considerable heterogeneity. We subsequently calculated areas under summary receiver operating characteristic curves (SAUROC), and we used plots observed data in ROC plane to assess threshold effect visually. In addition, we used graphical model tests to depict both the residual-based goodness-of-fit and the bivariate normal distribution, to check for how observations influenced analyses and to detect outliers.

Finally, we performed a meta-regression to explore how individual and pooled parameters included in the algorithms influenced the performance of automated algorithms for CLABSI/CRBSI detection as compared to manual surveillance.

We used the “midas” command (meta-analysis integration of diagnostic test accuracy studies), a comprehensive program of statistical and graphical routines for undertaking meta-analysis of diagnostic test performance in STATA developed by Dwamena [21]. Each individual parameter and combination of parameter were included in the meta-regression as an independent explanatory variable. We considered results as significant for P-values < 0.05. We used STATA/MP software (version 16.0). STATA codes are reported in the Additional file 1.

## Results

### Systematic literature search

We identified 586 non-redundant study records. Eighty (13.7%) full text articles were assessed for eligibility after abstract and title screening (Additional file 1: Fig. S1). Of these, 5 (1%) were included in the systematic review and meta-analysis [22–26]. Details on the search strategy are given in the Additional file 1.

### Characteristics of included studies

Three of the studies included in the systematic review were monocentric [23–25] and the remaining two multicentric [22, 26] (Table 1). Four studies analyzed only central venous catheters (CVC) and used CLABSI as an outcome [22, 24–26]. One study included unspecified IVC and used CRBSI as an outcome [23]. Two studies (40%) were conducted in the ICU setting [24, 26]. All studies were observational and used manually collected surveillance data as reference [22–26].

**Table 1:** Characteristics of included studies

Study	Setting	Type of ward	Location	Study period	Study population sample size	Catheter types included	Outcome
Trick et al. [22]	2 hospitals	All wards excluding neonatal and pediatric wards	US, Chicago	Sep 1st, 2001 to Feb 28th, 2002	99 patients (104 positive blood culture) in one hospital, and 28 patients (31 positive blood culture) in the other hospital	CVC	CLABSI
Bellini et al. [23]	1 hospital	All types	Switzerland, Lausanne	3-years period	669 positive blood culture	Unspecified intravascular catheter	CRBSI
Woeltje et al*.* [24]	1 hospital	6 ICU	US, Missouri	July 1st, 2005 to Dec 31, 2006	540 patients (694 positive blood culture)	CVC	CLABSI
Woeltje et al*.* [25]	1 hospital	4 non-ICU	US, Missouri	Jul 1st, 2005 to Dec 31, 2006	331 patients (391 positive blood culture)	CVC	CLABSI
Snyders et al*.* [26]	11 hospitals	17 ICU	US, Missouri	Jan1st to Jun 30, 2011	518 patients (643 positive blood culture)	CVC	CLABSI

CVC: central venous catheter; CLABSI: central line-associated bloodstream infection; CRBSI: catheter-related bloodstream infection ICU: intensive care unit

Across the 5 studies, 32 different automated algorithms or population samples were identified and included in the meta-analysis. Among the 32 algorithms, we identified 7 individual parameters, and 9 combinations of parameters used for automated detection of CLABSI/CRBSI (Table 2 and Additional file 1: Table S1). These 16 single or pooled parameters were then tested in the univariable meta-regression.

**Table 2:** Individual and pooled parameters used in the meta-regression

Individual or pooled parameters	Definitions	Corresponding letter in Fig. 2
Hospital-acquired (HA) bloodstream infection (BSI) with IVC	Hospital-acquired BSI was defined as a BSI detected ≥ 48 h after hospital admission and the patient had an intravascular catheter in situ	A
True BSI for common skin commensal	True BSI for common skin commensal (CSC) was defined as at least two positive blood cultures within 3 to 7 days according to studies included. CSC included *diphtheroid*, *Bacillus* species, *Cutibacterium* species, coagulase-negative staphylococci, and micrococci	B
Clinical data	Clinical data (e.g., fever defined by temperature > 38.0 °C, hypotension defined by systolic pressure < 90 mmHg) were considered in the algorithm	C
Administration of antibiotics	Administration of antibiotics was considered in the algorithm	D
New episode	The same microorganism isolated in a separate blood culture was considered as a new episode only if identified after at least between 3 and 7 days after the first episode, according to studies	E
Cultures from any other sites	A bloodstream infection was not considered catheter related or associated if the same pathogen was identified from a culture in any other body sites	F
Cultures from specific other sites	A bloodstream infection was not considered catheter related or associated if the same pathogen was identified in cultures from one of the following body sites: respiratory track, urinary or wound	G
HA BSI with IVC + true BSI for common skin commensal + clinical data	Cf. definitions above	H
HA BSI with IVC + true BSI for common skin commensal + administration of antibiotics	Cf. definitions above	I
HA BSI with IVC + true BSI for common skin commensal + new episode	Cf. definitions above	J
HA BSI with IVC + true BSI for common skin commensal + culture from any other sites	Cf. definitions above	K
HA BSI with IVC + true BSI for common skin commensal + culture from specific another sites	Cf. definitions above	L
HA BSI with IVC + true BSI for common skin commensal + new episode + culture from any other sites	Cf. definitions above	M
HA BSI with IVC + true BSI for common skin commensal + new episode + culture from specific another sites	Cf. definitions above	N
HA BSI with IVC + true BSI for common skin commensal + clinical data + culture from any other sites	Cf. definitions above	O
HA BSI with IVC + true BSI for common skin commensal + clinical data + culture from specific another sites	Cf. definitions above	P

BSI: bloodstream infection; IVC: intravascular catheter; HA: healthcare associated; CSC: common skin commensal

### Quality of studies

Using the QUADAS-2 tool, the quality of the five studies included in the systematic review and meta-analysis was assessed to be high (Additional file 1: Table S2). Overall, the risk of bias was low: two studies were rated as *low* risk of potential bias, among all assessed categories [22, 24], and three studies were rated as *high* risk of bias for only one algorithm within each study [23, 25, 26]. The applicability was rated as *high* for four of the five studies [22, 24–26], and only one study had *low* applicability in two of three algorithms identified in the study [23]. The evaluation of publication bias is illustrated in Additional file (Additional file 1: Figs. S2 and S3).

### Pooled sensitivity, specificity and SAUROC

**Fig. 1:**
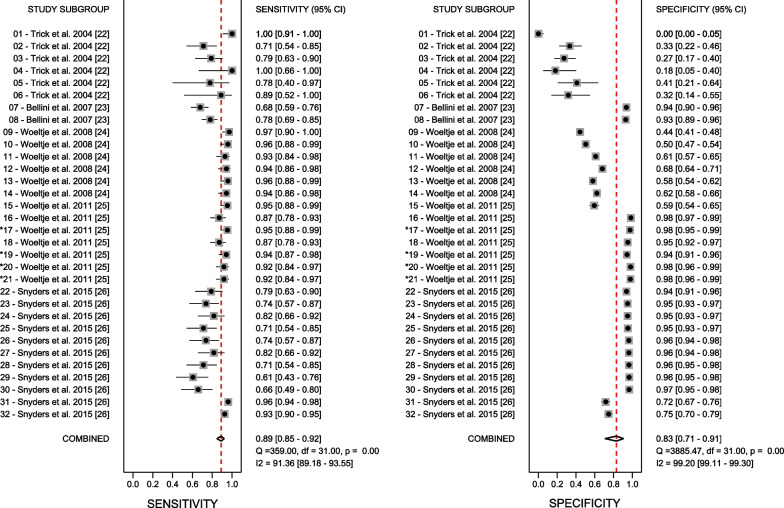
Forest plot of diagnostic performance, including sensitivity and specificity. *Studies with the best performance based on both sensitivity/specificity (sensitivity ≥ 0.89 and specificity ≥ 0.83)

The pooled sensitivity and specificity were 0.89 [95% CI 0.85–0.92] and 0.83 [95% CI 0.71–0.91] with significant heterogeneity between algorithms included in the meta-analysis (*I*^2^ = 91.36, *p* < 0.001 and *I*^2^ = 99.20, *p* < 0.001), respectively (Fig. 1).

The area under the SAUROC curve was 0.93 [95% CI 0.91–0.95] and this identified four algorithms with sensitivity greater than 0.89 and specificity greater than 0.83 (Figure S4A). These 4 best performing algorithms were all from the study of Woeltje et al*.* 2011 [26]. Algorithm 17 was defined by the combination of the four following parameters: “*Hospital-acquired BSI with CVC*”, “*True BSI for common skin commensal (CSC)*”, “*Clinical data”* and “*Cultures from any other site”* and has a sensitivity of 0.95 [95% CI 0.88–0.99] and a specificity of 0.98 [95% CI 0.95–0.99]. Three algorithms (19, 20 and 21) combined the same three parameters “*Hospital-acquired BSI with CVC”,* “*True BSI for CSC”* and “*Cultures from any other site”* and differed regarding the time window considered for the parameter “*Cultures from other body sites”* (from admission to the positive blood culture (algorithm 19; sensitivity 0.94 [95% CI 0.87–0.98] and specificity 0.94 [95% CI 0.91–0.96]) *or* from admission and within 7 days before or after the positive blood culture (algorithm 20; sensitivity 0.92 [95% CI 0.84–0.97] and specificity 0.98 [95% CI 0.96–0.99]) *or* from admission and within 14 days before to 7 days after the positive blood culture (algorithm 21; sensitivity 0.92 [95% CI 0.84–0.97] and specificity 0.98 [95% CI 0.96–0.99]).

Outliers regarding sensitivity and specificity are illustrated in the Additional file (Additional file 1: Fig. S2).

### Meta-regression

**Fig. 2:**
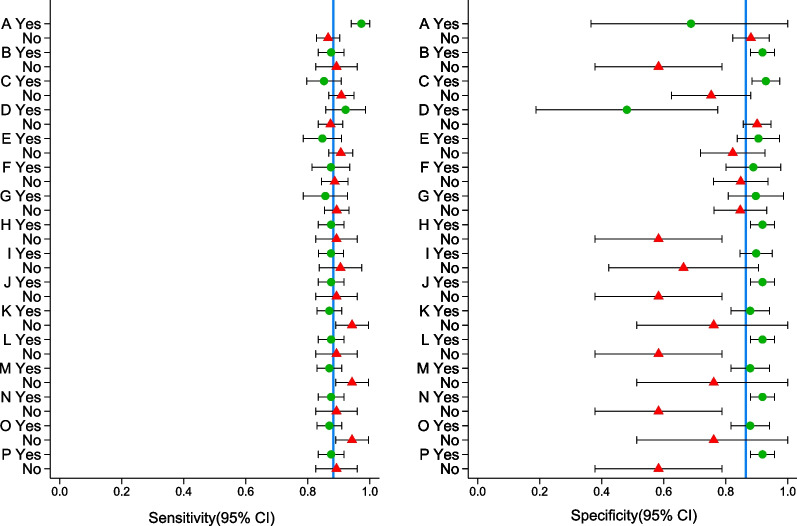
Univariable meta-regression for intravascular catheter bloodstream infection criteria. Blue reference line shows the pooled sensitivity and specificity, respectively. Letters A to G refers to Individual and pooled parameters described in Table 2

Given the heterogeneity between algorithms included in the meta-analysis, the meta-regression sought to identify which parameters had the greatest influence on the measures of effect. Additional file 1: Fig. S5 shows the frequency of all individual and pooled parameters identified within the 32 algorithms. The individual and pooled parameters for CLABSI/CRBSI detection derived from univariable meta-regression is illustrated in Fig. 2.

**Fig. 3:**
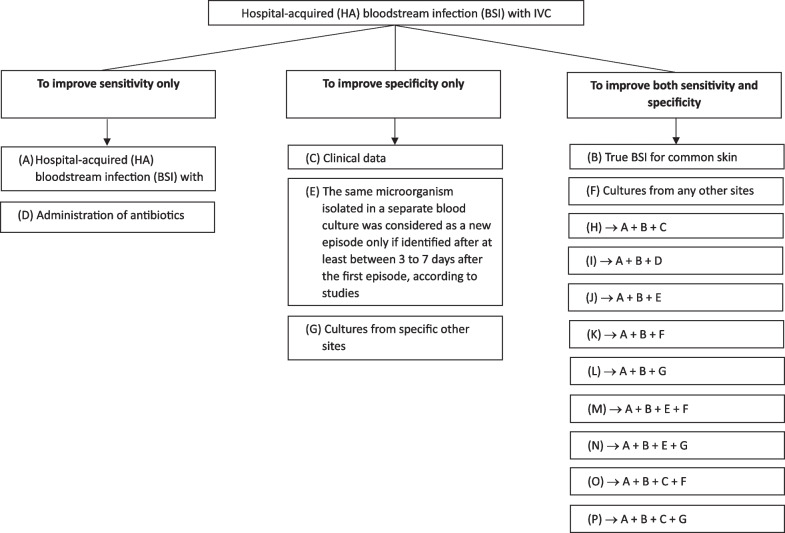
Decision tree based on different algorithm’s parameters

Figure 3 summarizes how each parameter affects the sensitivity and specificity of the algorithm. For two parameters only, *“Hospital-acquired BSI with CVC”* and *“Administration of antibiotics”*, the sensitivity increased, and the specificity decreased when the parameter was present. When *“Hospital-acquired BSI with CVC”* was the only parameter considered, the sensitivity was 0.98 (95% CI 0.96–1.00) as compared to 0.83 (95%CI 0.83- 0.90) when it was not considered (*p* = 0.25). When *“administration of antibiotics”* was the only parameter considered, the sensitivity was 0.92 (95% CI 0.85–0.99) as compared to 0.88 (95% CI 0.84–0.92) when it was not considered. For all other individual and pooled parameters, the sensitivity decreased, and the specificity increased when the parameter was present.

The combination of parameters with the highest specificities always included the parameter *“True BSI for common skin commensal”.* In addition, combination with highest specificities included either *“Culture from specific other sites”* or *“Culture from any other site”* and either *“New episode”* or *“Clinical data”.* The combinations that included the parameter *“Culture from specific other sites”* had higher specificities (0.92; 95% CI 0.88–0.96) than the combinations that include *“Culture from any other site”* (0.88; 95% CI 0.81–0.95). The addition of the parameter *“Clinical data”* in the combination did not increase the specificity of the estimate: 0.92 (95% CI; 0.88–0.96) with the parameter included *vs*. 0.92 (95% CI; 0.88–0.96) without it.

## Discussion

To our knowledge, this is the first meta-analysis that has reported pooled sensitivity and specificity as predictive performance of automated surveillance algorithms, using bivariate random-effects approach from 5 studies published in the last two decades [27–30]. This meta-analysis highlighted two main findings: (1) the pooled sensitivity was high but heterogeneous across all algorithms; and (2) the pooled specificity was also heterogeneous, but the meta-regression allowed to identify several individual and pooled parameters that had a greater influence on the measure of effect, and which could therefore inform the development of further automated surveillance algorithms for the detection of CLABSI/CRBSI.

Few studies have investigated the predictive accuracy of both CLABSI and CRBSI using automated surveillance systems. Frequently, authors have focused on CVC and CLABSI, thus disregarding other IVC and the more specific definition CRBSI. Indeed, the CRBSI definition frequently needs catheter removal and catheter tip culture, which are not commonly performed in most countries [31]. However, CRBSI allows a higher degree of certainty in the attribution of the catheter in the occurrence of the BSI [17], as compared to the CLABSI definition. To our knowledge, only one previous systematic review collated data on automated surveillance algorithms for the detection of IVC infections [27]. However, the authors of this systematic review focused on all HAIs, did not perform a meta-analysis and did not assess heterogeneity in the definitions used for automated surveillance of IVC infections.

In our study the pooled sensitivity was high (i.e., > 85%), and no individual or pooled parameter substantially influenced it. This finding is probably explained by the simplicity of detection of BSI compared to other HAIs (e.g*.* surgical site infections or ventilator-associated pneumonias) that require more complicated detection strategies and inclusion of clinical data or radiological findings.

Several factors influenced specificities and could improve the accuracy of automated monitoring algorithms. First, the addition of “clinical signs” did not substantially improve the accuracy of the specificity compared to similar algorithms that included microbiological cultures from other body sites. Second, higher specificities could be achieved by including only microbiological data from respiratory tract, urinary tract or wound samples instead of microbiological data from any other body sites. Clinical signs are frequently difficult to collect in hospital databases because of the lack of structure and standardization of the data, whereas microbiological cultures, which usually rely only on microbiological laboratories, are often more harmonized, more easily extractable and therefore integrated in an automated surveillance algorithm. Automated algorithms should firstly integrate microbiological data from other selected body sites to exclude non-catheter associated bloodstream infections.

Our results allow us to make some suggestions regarding which parameters are relevant for clinical decision making or hypothesis generation for further clinical studies investigating this issue, based on the balance between sensitivity and specificity and on clinical relevance (Fig. 3). We suggest that the following parameters should be relevant for inclusion in an automated CLABSI detection algorithm: (i) a parameter to differentiate a common skin contaminant from a true causative pathogen; (ii) a parameter to define two distinct infectious episodes; and (iii) a parameter to consider cultures from other specific body sites to exclude infection not associated with an IVC. That is, our results allow us to infer that any automated algorithm for IVC bloodstream surveillance needs to be able to: distinguish the causative pathogen from common skin commensal; consider the same microorganism isolated in a separate blood culture to be a new infectious episode if identified after at least between 3 and 7 days after the first episode; and differentiate any positive culture(s) from other body sites as being non-catheter related.

The use of a meta-regression versus predictive scores (e.g., Infection Probability Score (IPS) [32], or its modification for central venous catheter-related bloodstream infections [33]) remains debated. While the development and validation of predictive score are usually based one restricted and temporally defined sample (i.e., study), systematic reviews, meta-regression and meta-analysis include several samples (i.e., several clusters, representing different studies). Predictive scores can be validated externally in other settings, allowing model discrimination and calibration performance across several settings. However, random-effects meta-regression or meta-analysis provide the additional benefit of average performance and heterogeneity in performance across different studies. Accordingly, systematic reviews, meta-regression and meta-analysis of validation studies provide a summary of predictive performance from different settings and populations [34]. In other words, meta-regressions or meta-analysis provide a potentially more accurate and relevant measure of performance, taking into account context and heterogeneity factors. This makes meta-regressions and meta-analysis a more relevant tool for decision making purposes taking into account the external validation of an automated algorithm.

This study has some limitations. First, the low number of studies included suggests that the accuracy of the estimates of automated monitoring algorithms for CLABSI/CRBSI detection could be limited by the potential heterogeneity of data. Moreover, the inclusion of Bellini et al. [23] study, which relied on CRBSI definition only, could have increased the risk of heterogeneity. Second, classification of algorithms could be questionable, and the heterogeneity in definitions did not allow us to develop accurate algorithms (e.g., details of clinical signs were not specified, time-windows were simplified into our meta-analysis by regrouping time-windows from different studies). Third, the generalizability of our conclusions could be limited, because studies were frequently monocentric and were all performed in high income countries. Fourth, grey literature was not screened. Finally, mostly CVC were included; it is conceivable that our results are not applicable to other intravascular catheter types.

## Conclusions

Our meta-regression examined the accuracy of automated algorithms developed to monitor CLABSI/CRBSI and provides valuable information while developing valid algorithms for automated monitoring of intravascular catheter infections. Microbiological cultures from selected other body sites could help to exclude BSI not related to IVC, whereas clinical signs did not substantially improve the accuracy of automated systems when microbiological cultures from selected other body sites were included.

### Supplementary Information

**Additional file 1**. Search strategy, Supplementray figures and tables.

## Data Availability

Data and materials are available in the text of the article and in Supplementary material. JMJ had full access to all of the data in the study and takes responsibility for the integrity of the data and the accuracy of the data analysis.
