# Supplementary material for: Predictive performance of automated surveillance algorithms for intravascular catheter bloodstream infections: a systematic review and meta-analysis
Source: Antimicrob Resist Infect Control. 2023 Aug 31;12:87. doi: 10.1186/s13756-023-01286-0 (PMC10468855; doi:10.1186/s13756-023-01286-0)
Supplement: Supplementary file 1 — Additional file 1. Search strategy, Supplementray figures and tables. [file 13756_2023_1286_MOESM1_ESM.docx]

**Supplementary materials**

**Search strategy**

**Key-words algorithm used in PubMed** (https://pubmed.ncbi.nlm.nih.gov/)

((((catheter[Title/Abstract]) AND ((infect*[Title/Abstract]) OR (bact*[Title/Abstract]))) AND (((automat*[Title/Abstract]) OR (electronic*[Title/Abstract])) OR (comput*[Title/Abstract]))) AND ((surveil*[Title/Abstract]) OR (monitor*[Title/Abstract])) AND (2000/1/1:2021/12/31[pdat]))

OR

((((hospital acquir*[Title/Abstract]) AND ((infect*[Title/Abstract]) OR (bact*[Title/Abstract]))) AND (((automat*[Title/Abstract]) OR (electronic*[Title/Abstract])) OR (comput*[Title/Abstract]))) AND ((surveil*[Title/Abstract]) OR (monitor*[Title/Abstract])) AND (2000/1/1:2021/12/31[pdat]))

**Key-words algorithm used in EMBASE** (https://www.embase.com/)

catheter:ab,ti AND ('infect*':ab,ti OR 'bact*':ab,ti) AND ('automat*':ab,ti OR 'electronic*':ab,ti OR 'comput*':ab,ti) AND ('surveil*':ab,ti OR 'monitor*':ab,ti) AND [1-1-2000]/sd NOT [1-1-2022]/sd

OR

'hospital acquir*':ab,ti AND ('infect*':ab,ti OR 'bact*':ab,ti) AND ('automat*':ab,ti OR 'electronic*':ab,ti OR 'comput*':ab,ti) AND ('surveil*':ab,ti OR 'monitor*':ab,ti) AND [1-1-2000]/sd NOT [1-1-2022]/sd

**Search algorithm for high sensitivity**

**(HAI identification)**

**Search algorithm for high specificity (intravascular catheter infection identification)**

+111

+212

PubMed Records n=175

EMBASE records, n=200

**Identification**

Duplicated from EMBASE, n=89

Non-redundant records, n=226

Duplicated from EMBASE, n=152

Non-redundant records, n=387

EMBASE records n=364

PubMed records, n=115

75 full-text articles excluded:

- 3 did not report automated or semi-automated surveillance of CLABSI/CRBSI
- 2 did not report data for CLABSI/CRBSI
- 30 did not reported validation using diagnostic test method
- 34 were conference abstracts
- 1 review
- 2 articles in Turkish
- 1 not available full text
- 1 study evaluating semi-automated algorithm
- 1 study evaluating automated extraction of denominator data

505 records excluded:

- 488 did not report automated surveillance of CLABSI/CRBSI
- 1 without abstract available
- 5 conference abstracts
- 11 did not report original studies

Full-text articles assessed for eligibility, **n=80**

+199

Records after exclusion of duplicates **n=586**

Duplicated from ALGO#2, n=27

**Screening**

**Eligibility**

**Included**

Studies includes for quantitative analysis, **n=5**

**Figure S1.** Flow-chart.

HAI: Hospital acquired infection; CLABSI: Central line bloodstream infection; CRBSI: Catheter-related bloodstream infection

STATA Code for meta-analysis using the ‘midas’ (meta-analysis integration of diagnostic test accuracy studies) command, developed by Dwamena BA [21].

*** Meta-analysis for CLABSI Automated Surveillance Algorithms Accuraccy ***

* Summary Statistics *

midas tp fp fn tn, res(all)

* Table of index-specific results *

midas tp fp fn tn, table(dlr)

* Forest plot to demonstrate study-specific on right y-axis *

midas tp fp fn tn, id(id_study0) ms(0.75) ford fors bfor(dss)

* Summary ROC Curve with prediction and confidence Contours *

midas tp fp fn tn, plot sroc(both)

* Linear regression test of funnel plot asymmetry *

midas tp fp fn tn, pubbias

* Bivariate Boxplot *

midas tp fp fn tn, bivbox scheme(s2color)

* Quality Assessment *

midas tp fp fn tn, qbar(A B C D E F G H I J K L M N O P) qlab

* Meta-regression *

midas tp fp fn tn, reg (A B C D E F G H I J K L M N O P

| **Parameter** | **Definitions** | **Studies** | | | | |
| --- | --- | --- | --- | --- | --- | --- |
|  |  | Trick et al. 2004 [22] | Bellini et al. 2007 [23] | Woeltje et al. 2008 [24] | Woeltje et al. 2011 [25] | Snyders et al. 2015 [26] |
| HA-BSI with CVC | Baseline group was defined as a hospital-acquired BSI ≥48 hours after hospital admission) and an intravascular catheter in situ | 1*, 2, 3, 4*, 5, 6 |  | 9*, 10, 11, 12, 13, 14 | 15*, 16, 17, 18, 19, 20, 21 | 22, 23, 24, 25, 26, 27, 28, 29, 30, 31, 32 |
| True BSI for common skin commensal | True BSI for common skin commensal (CSC) was defined as at least two positive blood cultures within 3 to 7 days according to studies included. CSC included diphtheroid, *Bacillus* species, *Propionibacterium* species, coagulase-negative staphylococci, and micrococci. |  | 7, 8 | 10, 11, 12, 13, 14 | 16, 17, 18, 19, 20, 21 | 22, 23, 24, 25, 26, 27, 28, 29, 30, 31, 32 |
| Clinical data | Clinical data (e.g., fever defined by temperature >38.0°C, hypotension defined by systolic pressure <90 mmHg) were considered in the algorithm |  |  | 10, 14 | 16, 17, 18 | 22, 23, 24, 25, 26, 27, 28, 29, 30, 31, 32 |
| Antibiotics | Antibiotics were considered in the algorithm | 3, 6 |  | 11, 13, 14 |  |  |
| New episode | The same microorganism was considered as a new episode only if identified after at least between 3 to 7 days after the first episode, according to studies |  |  | 12, 13, 14 |  | 22, 23, 24, 25, 26, 27, 28, 29, 30, 31, 32 |
| Cultures from other body site | A bloodstream infection was not considered if the same pathogen was identified from another positive culture in any other body site | 2, 3, 5, 6 |  |  | 16, 17, 18, 19, 20, 21 | 23, 26 |
| Cultures from restricted other body sites | A bloodstream infection was not considered if the same pathogen was identified in the following body sites: respiratory tract~~k~~, urinary or wound |  |  | 12, 13, 14 |  | 24, 25, 27, 28, 29, 30, 31 |

**Table S1.** Parameters used for CLABSI/CRBSI definition in the 5 included studies

* These algorithms were based on a single parameter.

|  | **Risk of bias^a^** | | | | **Applicability concerns^f^** | | |
| --- | --- | --- | --- | --- | --- | --- | --- |
| Study | Patient selection^b^ | Index test^c^ | Reference standard^d^ | Flow and Timing^e^ | Patient selection^g^ | Index test^h^ | Reference standard^i^ |
| Trick et al. 2004 [22] | Low | Low | Low | Low | High | High | High |
| Bellini et al. 2007 [23] | Low | Low | High (regarding CLABSI) | Low | Low (regarding CLABSI) | Low (regarding CLABSI) | High |
| Woeltje *et al.* 2008 [24] | High | Low | Low | Low | High | High | High |
| Woeltje *et al.* 2011 [25] | High | Low | Low | Low | High | High | High |
| Snyders *et al.* 2015 [26] | High | Low | Low | Low | High | High | High |

**Table S2**. Quality assessment of studies using QUADAS-2

^a^ Assessing potential risk of bias concerns comprised 3 sections: information used to support the judgment of risk of bias, signaling questions, and judgment regarding risk of bias, for the four domains respectively. We performed rating using a categorical variable defining 3-levels of quality assessment (low; high; unclear) for risk of bias. Low rating was interpreted as good quality and high rating as poor quality regarding the meta-analysis concern. In addition, the “unclear” rating was only when insufficient data are reported.

According to the four domains concerned, judgement for rating was based on the following questions, including sub-topics questions:

^b^ The main question was “could the selection of patients have introduced bias in diagnostic accuracy regarding the systematic review objective?” including the three following sub-topics: 1) was a consecutive or random sample of patients enrolled? 2) was a case–control design avoided? and 3) did the study avoid inappropriate exclusions? – in particular, we rated high risk of bias if the study population target was not represented by all potential hospitalized patients (e.g., only ICU patients included).

^c^ The main question was “could the conduct or interpretation of the index test have introduced bias regarding the systematic review?” including the two following sub-topics: 1) were the index test results interpreted without knowledge of the results of the reference standard? and 2) if a threshold was used, was it prespecified?

^d^ The main question was “could the reference standard, its conduct, or its interpretation have introduced bias regarding the systematic review?” including the two following sub-topics: 1) is the reference standard likely to correctly classify the target condition? and 2) were the reference standard results interpreted without knowledge of the results of the index test?

^e^ The main question was “could the patient flow have introduced bias regarding the systematic review?” including the three following sub-topics: 1) was there an appropriate interval between the index test and reference standard? 2) did all patients receive the same reference standard? and 3) were all patients included in the analysis?

^f^ Applicability section was structured similarly to that of the risk of bias but do not include signaling questions. Judgement about applicability were rated as “low,” “high,” or “unclear", with inversed interpreting than the risk of bias section, e.g., low applicability displayed poor quality and high applicability displayed good quality regarding the meta-analysis concern.

**Figure S2.** Bivariate boxplot

Outliers identified by #2, 3, 5, 6, and 17 correspond to the following sub-groups into the meta-analysis: **2-**Trick et al. 2004 (b) [22], **3-**Trick et al. 2004 (c) [22], **5-**Trick et al. 2004 (e) [22], **6-**Trick et al. 2004 (f) [22], and **17-**Woeltje et al. 2011 (c) [25]) respectively.

**Figure S3.** Deeks’ funnel plot asymmetry test

**Figure S4.** Summary receiver operating characteristic (SAUROC) curve with prediction and confidence intervals.

Algorithms 17, 19, 20, and 21, located in the highest left corner represent the best performing algorithms defined by both sensitivity ≥0.89 and specificity ≥0.83.

**Figure S5.** Distribution of algorithms defined by single and pooled parameters.

Because single parameters might be in several algorithms, total the distribution was higher than 100%.

Algorithms were defined according single and multiple parameters described in table 2
